# Tuning adhesion forces between functionalized gold colloidal nanoparticles and silicon AFM tips: role of ligands and capillary forces

**DOI:** 10.3762/bjnano.9.61

**Published:** 2018-02-20

**Authors:** Sven Oras, Sergei Vlassov, Marta Berholts, Rünno Lõhmus, Karine Mougin

**Affiliations:** 1 Institute of Physics, University of Tartu, W. Ostwaldi tn 1, 50412, Tartu, Estonia; 2 Université de Strasbourg, Université de Haute Alsace, Institut de Science des Matériaux, IS2M-CNRS-UMR 7361, 15 Rue Jean Starcky, 68057 Mulhouse, France; 3 Department of Physics and Astronomy, University of Turku, FIN-20014 Turku, Finland

**Keywords:** adhesion, AFM, gold, nanoparticle functionalization, nanoparticles

## Abstract

Adhesion forces between functionalized gold colloidal nanoparticles (Au NPs) and scanning probe microscope silicon tips were experimentally investigated by atomic force microscopy (AFM) equipped with PeakForce QNM (Quantitative Nanoscale Mechanics) module. Au NPs were synthesized by a seed-mediated process and then functionalized with thiols containing different functional groups: amino, hydroxy, methoxy, carboxy, methyl, and thiol. Adhesion measurements showed strong differences between NPs and silicon tip depending on the nature of the tail functional group. The dependence of the adhesion on ligand density for different thiols with identical functional tail-group was also demonstrated. The calculated contribution of the van der Waals (vdW) forces between particles was in good agreement with experimentally measured adhesive values. In addition, the adhesion forces were evaluated between flat Au films functionalized with the same molecular components and silicon tips to exclude the effect of particle shape on the adhesion values. Although adhesion values on flat substrates were higher than on their nanoparticle counterparts, the dependance on functional groups remained the same.

## Introduction

Adhesion is a complex combination of various interfacial forces such as capillary, electrostatic and vdW forces. Depending on the particular situation, adhesion may represent either desirable or undesirable phenomena [1]. For applications like gluing, a strong adhesion is required to stick two surfaces together. On the other hand, for dynamical applications like sliding and rolling machinery, a strong adhesion may result in additional energy losses and wear at the interface. When it comes to the nanoscale, high adhesion can completely prevent the fabrication or functioning of micro- and nanoelectromechanical systems (MEMS and NEMS) with movable parts. Strong adhesion is necessary for keeping different parts of the micro- and nanodevices together without additional fixing procedures. Thus, the ability to tune adhesion between surfaces according to the particular needs is required to avoid failure or damage of the systems. Adhesion forces can be tuned according to particular application by proper surface treatment. One approach is the functionalization of the surfaces with various ligands. For instance, grafting a thin molecular film with hydrophobic tail groups on a surface, formed by self-assembly process, prevents the formation of nano- or microdroplets of water on the treated sample, or meniscus between two contact surfaces [2].

The real contact area is another important parameter that can be modulated to tune the adhesion between two surfaces [3–4]. A nice example at nano/microscale has been given by S. Casado [5], who shows how the modulation of roughness and adhesion of two different violin bow hairs observed at nanoscale by AFM, may cause strong consequences at mascoscopic scale during the stick–slip phenomenon of the rubbing hairs surfaces and in fine such different acoustic outputs. Therefore, control of the nanoscale interactions between two surfaces through chemistry and contact area is crucial for predicting and understanding the involved adhesion forces, which have a direct impact on the assembly of nano-blocks and the development of various nanomaterials.

Great interest towards the nanoscale materials and systems arises from their strongly enchanced and, in certain cases, completely unique properties, which already led to plently of applications in numerous fields of science [6] and technology including advanced materials for energy, health, food, space and military, as well as stronger, lighter, cleaner and “smarter” surfaces and systems.

Among the vast number of various nanoscale materials synthesised during the recent decades, Au nanostructures hold an especially important role. Au is one of the first materials that found applications in nanoscale form centuries ago, and still Au nanostructures continue to be the focus of intense studies due to inertness and high quality with low concentration of defects [7–8]. Moreover, Au nanostructures can be synthesized by relatively simple colloidal chemistry methods in various sizes and shapes with high degree of quality by varying the solution composition and relative reactant concentrations [9], which in turn allows for accurate control of their intrinsic properties. For instance, Au nanospheres, nanocubes, nanorods and nanowires [10] have been routinely synthesized. Their physico-chemical and especially optical properties [11] of NPs strongly depend on their shape, size and spatial arrangement [12–15]. All these make Au nanostructures a perfect model system for studying various physical and chemical properties, and provide opportunities to understand and develop new phenomena [7,16–17].

Au nanomaterials are usually deposited or grown on model substrates such as silicon wafer. Indeed, the most common substrate material for MEMS and NEMS is silicon. It can be tailored into complex shapes by fine lithography methods. The adhesion between Au and Si or SiO_2_ is known to be rather poor [18–20]. Böhme et al. [21] demonstrated that the oxygen layer forming on the silicon substrates affects the adhesion between Au particles and silicon substrates. Furthermore, Langbein et al. stated that the vdW attraction between two surfaces is only affected by their outer layers, if the separation of the surfaces is lower or in the same range as the thickness of the outer layer [22]. As a result, functionalization of either Au NPs or silicon substrates (or both) significantly and directly affects the adhesion of the particles and their mobility on the surface [19–20]. Hence, Darwich et al. have shown that the mobility of Au NPs was significantly impacted by the intermolecular interactions between an AFM tip and NPs, but also by the interactions of the NPs and silicon substrate during nanomanipulation in AFM in tapping mode [23]. Despite its inertness [24], Au NPs can be relatively easily functionalized with organic ligands resulting in the formation of stable colloids [23,25]. The possibility of changing the ligands chain length, tail group and the packing order (disorder) of the molecular coating makes functionalized NPs an attractive model systems for studying the nature of interactions, and particularly adhesion at the nano and molecular level. The prospect of modifying the chain length of the ligands, varying the end group molecule and the packing order of the functionalized NP makes it an appealing model system for studying intermolecular forces at a nanoscopic scale.

In this paper, we have investigated the effect of functionalization on the adhesion between Au NPs and sharp silicon tip by atomic force microscopy in ambient conditions. Au NPs were synthesized by chemical reduction of metal salts and functionalized with thiols presenting different tail groups varying from a very hydrophobic to a highly hydrophilic behavior. The tail groups were either an amino (–NH_2_), hydroxy (–OH), carboxyl (–COOH), methyl (–CH_3_), methoxy (–OCH_3_) or thiol (–SH). The adhesion was measured by atomic force microscope (AFM) equipped with a PeakForce QNM module. The results were also compared to additional adhesion measurements performed on flat Au films functionalized with the same molecular thin film to evaluate the impact of the NPs topography on the adhesion measurement at nanoscale.

## Results and Discussion

### Functionalized Au nanoparticles

**Figure 1:**
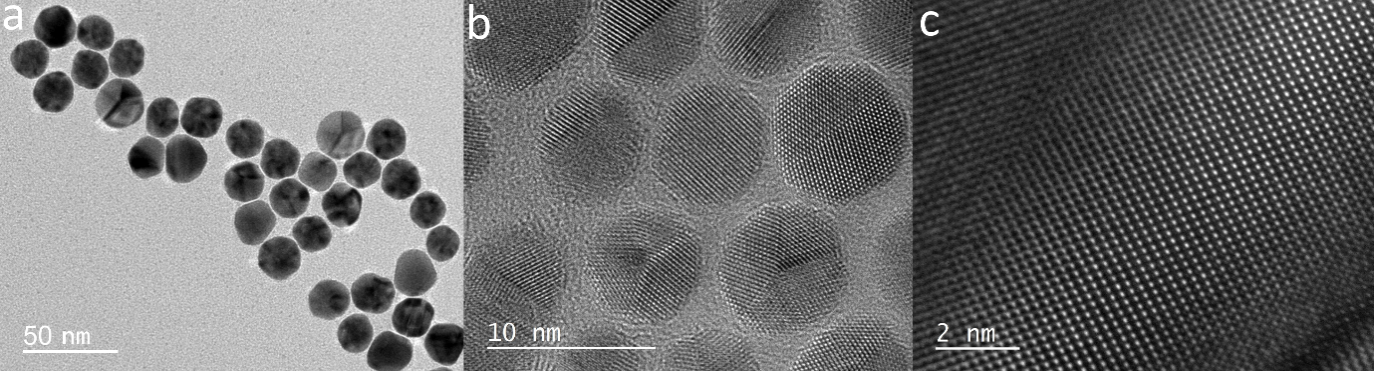
TEM images of Au NPs that tend to have a polyhedron-like shape a) 30 nm diameter Au NPs b) 5 nm diameter Au NPs c) zoom in on a NP to visualize the high organization of Au atoms in the NP.

Au colloidal NPs used in the study ranged from a few to several tens of nanometers in size and had a polyhedral structure with well-defined facets (Figure 1). The most common particle shapes were pentagonal dipyramids and octahedrons [26].

The synthesis of so-called “bare” Au NPs, described in more details in the experimental section, involved addition of sodium citrate [27] in order to prevent the agglomeration of the NPs in the solution. Sodium citrate molecules exchange only electrostatic interactions with Au atoms and residues of Au^3+^ ions. However, this interaction between Au NP and ligands is strong enough to provide a long-term stability of Au NPs in solution. In addition, it allows functionalization of the NPs with a partially hydrophilic thin coating. From this perspective, as-synthesized Au NPs can be also considered as functionalized because they are actually covered with sodium citrate stabilizing group (COO– or –COOH).

Further functionalization was carried out by molecule exchange procedure. As a result, functionalized NPs were coated with organosulfur (thiol) molecules having different tail groups ranging from a highly hydrophilic (–OH, –COOH, –SH, –NH_2_) to a partially hydrophilic (–O–CH_3_) and to a more hydrophobic group (–CH_3_). A covalent bond between the sulfur head group (–SH) and the Au atoms ensured the strong anchorage of the organosulfur molecules to the Au NPs. While the chemical interaction between the NPs and the thin molecular film is strong, its structural state may not be completely ordered. Indeed, the structural state of the thin ﬁlm is mainly defined by its uniformity and packing density. Granick and coworkers [28–29] demonstrated the importance of the surface and quantity of grafting sites to form well-packed monolayers of self assembled monolayers (SAMs) on a surface (or NPs), which will ensure a proper mechanical behavior and aging of the system and reduce its destabilization along the time. In addition, long chains of more than twelve carbons are usually self-assembled in a well-ordered structure referred to as a solid-like structure. For the long chains, the molecular layers are compact and rigid, for the short chains the layers are disordered in the thin molecular film. This might be explained by the strong intermolecular interactions that hold large blocks of molecules together and allow stabilization of the thin film by vdW attractions formed between the molecule chains [30–31]. Thus the structural state of the thin ﬁlm and the packing density represent important intrinsic parameters of the thin NPs coating that have strong influence on the intermolecular interactions between the silicon tip and the Au NPs.

### Adhesion of Au colloidal nanoparticles

**Figure 2:**
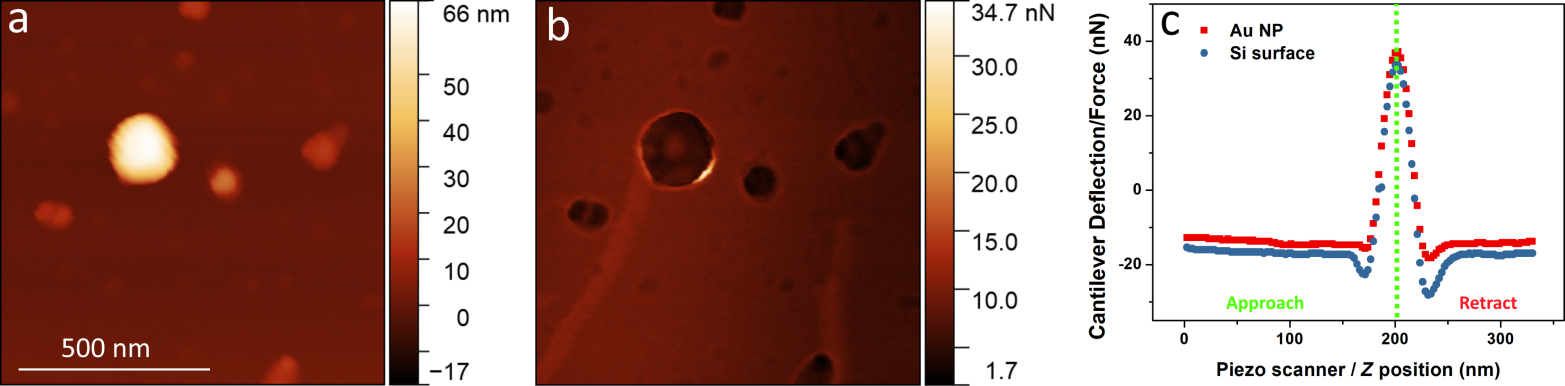
a) AFM-QNM topography image of colloidal Au NPs. b) AFM-QNM adhesion image of colloidal Au NPs. c) Force–distance curves for Au particle (red) and Si surface (blue).

Freshly prepared NPs were drop-casted onto a silicon wafer and dried as described in the experimental section. Topography and adhesion of the NPs were measured by AFM in a PeakForce QNM Mode. Figure 2 displays AFM images of Au NPs topography and adhesion along with typical force–distance curves for Au NPs and Si substrate.

Results show that adhesion force values are highly modulated by the nature of the tail group of the NPs thin coating as well as the NPs diameter. At constant NPs diameter, adhesion decreases in the following sequence: –SH, –NH_2_, –OCH_3_, –OH, –COOH and –CH_3_.

**Figure 3:**
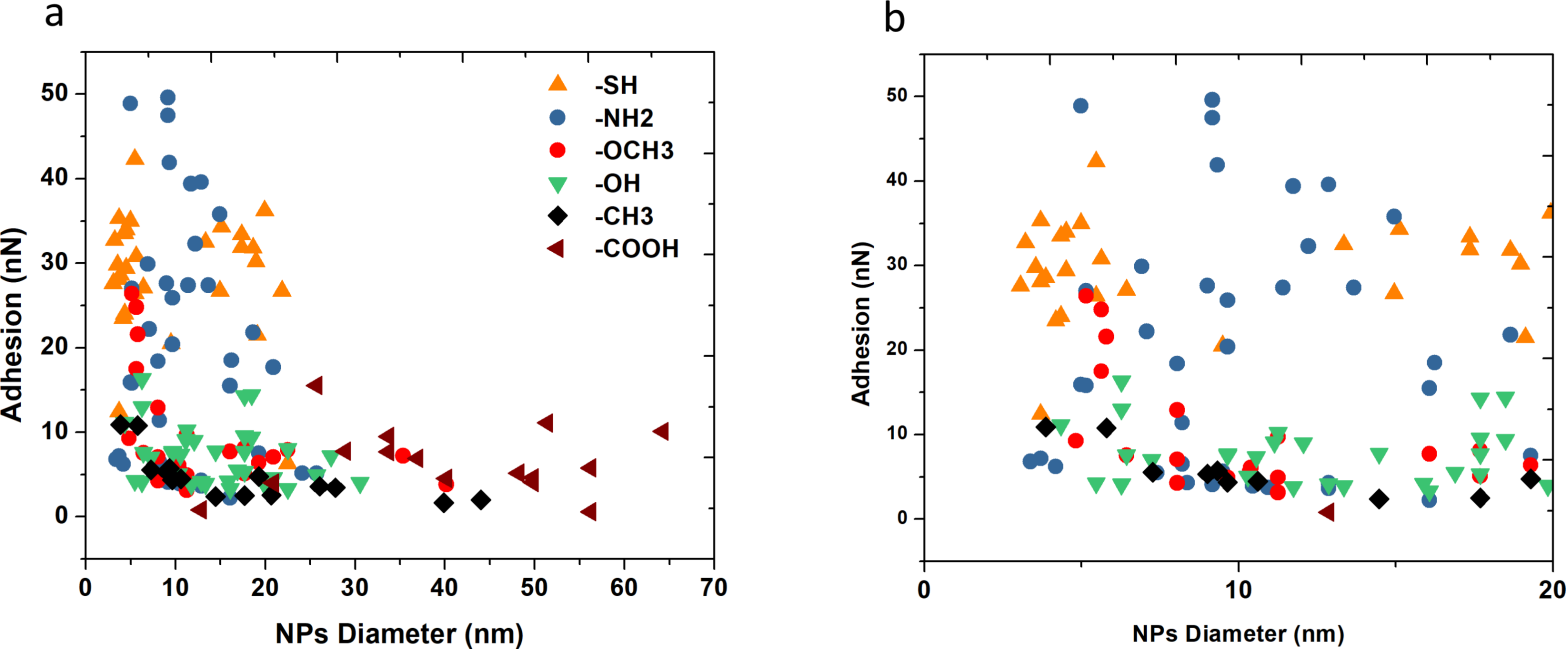
a) Adhesion values between silicon tip and functionalized Au NPs measured with Peakforce QNM AFM as a function of Au NPs diameter and functionalization: orange triangle –SH, blue circle –NH_2_, red circle –OCH_3_, green triangle –OH, black diamond –CH_3_, brown triangle –COOH end-groups; b) zoom in on NPs with diameters below 20 nm.

To explain these results, a combination of several intermolecular interactions and phenomena influencing adhesion should be considered. Factors such as vdW or dipole-induced forces, electrostatic forces, H-bonding and capillary forces are crucial to understand the dependence of adhesive force on the type of functional layer. First, the high adhesion values of –SH and –NH_2_ coated NPs are explainable by the high polarizability of the molecules. Hence, adhesion forces acting between hydrophilic NPs (including –SH, –COOH, –OH and –NH_2_ tail groups) and the silicon probe seem to be controlled mainly by attractive forces such as electrostatic and H-bonding as well as capillary forces. For NPs size varying from 5 to 20 nm diameters, the range of adhesion values of –NH_2_ coated NPs is quite large varying from 10 up to 50 nN (Figure 3b). An identical behavior is observed for –SH coated Au NPs as adhesion values vary from 20 up to 40 nN. Unexpectedly, the adhesion forces of –OH coated NPs are rather low, ranging from 5 to 15 nN and showing identical behavior of ligands with a partially hydrophobic tail group. The spread of these values is relatively large, in terms of absolute values for small NPs. This behavior can be explained by the contribution of different attractive forces controlling the interactions between Au NPs and Si AFM sharp probe. Obviously, electrostatic and H-bonding interactions represent a major part of the molecular forces acting between both contact surfaces, however, the presence of water vapor (humidity rate of the order of 35%) in air, has a strong impact on these results via capillary forces. In ambient conditions a water mono-bilayer with thickness around 1 nm can be formed around the NPs and AFM tip according to Asay [32]. The form a liquid condensate takes around the tip–substrate contact area depends on the spreading coeffiecient of the system. The spreading coefficient between solid–liquid–air interfaces is given by

[1]
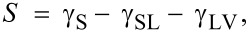


where γ_S_ is the interface energy of the bare solid, γ_SL_ is the interface energy between solid and liquid and γ_LV_ is the interface energy between liquid and vapour. This parameter shows the surface energy per unit area between the tip–liquid and substrate–liquid contacts.

In the presence of humidity, *F*_adh_ is the sum of the direct adhesion *F*_ss_ of the two contacting solids within the liquid, and the capillary force *F*_c_, due to the Laplace pressure of the water meniscus forming between the tip and the sample [33–34]:

[2]
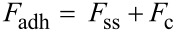


For a spherically shaped meniscus, *F*_c_ in first order by [35] can be written as

[3]
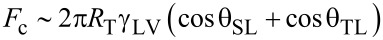


where *R*_T_ = 10 nm is the tip radius, θ_SL_ = 5° and θ_TL_ = 60° [36] are the static contact angles of the liquid on substrate (–NH_2_) and tip, respectively and γ_LV_ = 7.28 × 10^−2^ N/m is the liquid (water) surface tension. For the given values the capillary forces are around 7 nN.

From Equation 2, it can be deduced that for the low humidity there is no meniscus and *F*_adh_ = *F*_ss_. From a certain threshold humidity a single meniscus forms giving rise to a larger but constant adhesion force.

Although AFM tip in our experiments constantly moves relative to Au NPs, the velocities of the tip are not high enough to completely exclude the formation of capillary bridges between the tip and particle [37]. The AFM force-curves are conducted at a rate of 2 kHz allowing a contact of both surfaces in the order of the milliseconds [38–39] at ambient temperature (20 °C) and relative humidity around 35 ± 5%. A pure equilibrium state ensuring the constant formation of the water meniscus might be difficult to reach all the time as these values are at the formation limit, as discussed by Haugstad et al. [39]; however, it is still possible and it can explain the large spread of adhesion values obtained for –NH_2_ and –SH coated NPs. The capillary forces also depend on the size and shape of the particles, and longer contact times may contribute to the increase of these interaction forces. The low values of adhesion obtained for –OH coated Au NPs can be explained by a possible contamination of the Au NPs deposited on the silicon wafer. Due to the high polarity of the –OH functional group compared to those of –NH_2_ and –SH groups, a higher adhesion value might have been expected. However, this high polarity of the tail group also confers a high reactivity to Au NPs, especially with contamination in air. As a result, the final adhesive behavior of the Au coated NPs remains similar or slightly lower than hydrophobic coated NPs (5–10 nN) inducing molecular and long-range interactions of the same order. The distribution of the adhesion results is yet lower than for –SH and –NH_2_ coated NPs. –COOH coated Au NPs also have low adhesion values, independently of Au NPs size, varying from 20 up to 70 nm diameter. The carboxylic acid terminated Au NPs are surrounded by short chains organic molecules that confer a disordered SAMs coating with low surface density, compared to –OH tail group. For these raw NPs bearing residual stabilizing carboxy group, one can reasonably suppose that under ambient conditions an adsorbed thin water film exists at the NPs–substrate interface, that should increase the capillary forces and indirectly the adhesion between NPs and tip. On the other hand, high polarity of –COOH group increases the reactivity of NPs with airborne contaminants, which might hinder formation of water meniscus and prevent capillary forces or H-bonding.

**Figure 4:**
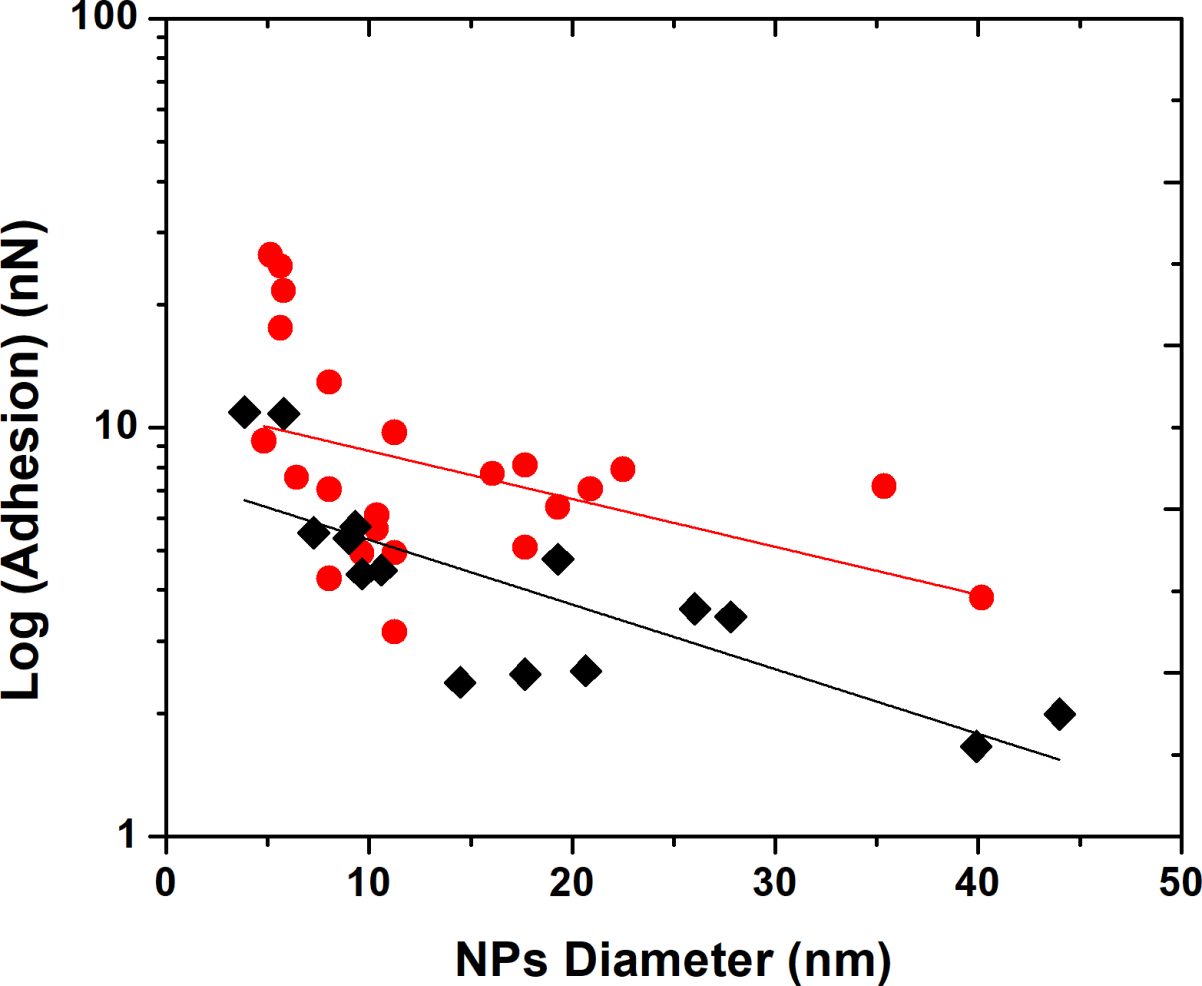
Logarithm of the adhesion between silicon tip and functionalized Au NPs deposited on silicon wafer measured with Peakforce QNM AFM, versus NPs diameters: red circles –OCH_3_, black diamonds –CH_3_. Fitting parameter *R*² is approx. 0.80 for both groups.

Contrary to NPs with a hydrophilic coating, hydrophobic (–CH_3_) and partially hydrophobic (–OCH_3_) coatings display a different behavior. Figure 3 indicates that the presence of a hydrophobic interface significantly reduces the adhesion values, being the smallest for the most hydrophobic NPs containing –CH_3_ tail group. Moreover, contrary to hydrophilic Au NPs, adhesion on hydrophobic NPs is strongly dependent on the NPs size as shown in Figure 3b and Figure 4.

When plotted on a logarithmic scale, adhesion values for hydrophobic NPs can be well approximated by linear fit corresponding to an exponential decay of the adhesion with NPs size, which is known as size effect [38]. This slope is slightly higher for the –CH_3_ coated NPs, indicating a stronger decrease of the input molecular interactions with the NPs size than for partially hydrophobic NPs as one could expect for the lowest nanoparticle coating adhesion. Surprisingly, this behavior is only visible for hydrophobic NPs. An explanation to this behavior may come at least partly from the capillary effects observed on the hydrophilic functional group coated NPs that might inhibit this behavior due to the presence of the adsorbed water layer at the surface of the NPs under ambient conditions. It should be noted that dependence of adhesion on particle size can be actually more complicated than just exponential, however detailed study of this phenomena is outside of the scope of the present study.

### VdW calculation

To validate our results, vdW force between the functionalized Au particles and AFM tip were calculated. The simulation of these dipole-induced forces involves a large number of molecular structure-specific variables. Therefore, only thiol as an example tail group was chosen, to estimate the order of magnitude of vdW interaction force and its contribution to overall adhesion forces.

VdW interaction forces for thiol molecules and silicon in contact was calculated according to Equation 3 [40]:

[4]
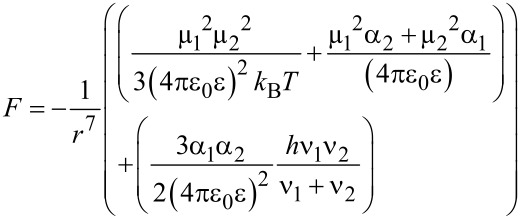


where µ_1_ = 7.2 × 10^−31^ C·m and µ_2_ = 1.0 × 10^−29^ C·m are the dipole moments, α_1_ = 6.8 × 10^−40^ C^2^·m^2^/J and α_2_ = 5.8 × 10^−40^ C^2^·m^2^/J are the polarizabilities, ν_1_ = 2.8 × 10^15^ Hz and ν_2_ = 2.1 × 10^15^ Hz are the ionisation frequencies of silicon and thiol respectivly, *k*_B_ is Boltzmann’s constant, *T* = 293 K is the temperature of the medium, ε_0_ is the vacuum permittivity, ε = 1 is the relative permittivity and *r* = 0.165 nm [33] is the typical cut-off distance. According to calculations, the average values of vdW force between the molecules are around 13 nN. This value allows validating AFM measurements (Figure 3b). Therefore, vdW forces remain one of the main interactions forces between NPs and Si tips, apart from other molecular interactions such as H-bonding and capillary forces.

### SAM packing density and adhesion

In addition to the previously discussed factors, packing density of SAM on the surface has also an important role in adhesion [41]. The self-organization and close packing of the molecules ensured by vdW interactions between chains plays an essential role in stabilizing thin molecular ﬁlms or coatings.

It is well known that molecules with short alkyl chains (*n* < 8, where *n* represents the number of carbon atoms groups along the chain skeleton of the molecules) self-assemble on a surface with rather a poor packing [2]. This kind of structuration leads to low density of molecules on the surface. As a result, short chains have more structural disorder and defects that promote energy dissipation through rotational and vibrational excitation modes [30]. These excitation modes contribute strongly to the energy transfer to the substrate and thus to its physico-chemical and mechanical properties resulting in decrease of adhesion forces during a contact between two interfaces. On the contrary, longer molecules (*n* > 8) self-assemble in a well-packed system with higher cohesive interactions between the chains.

**Figure 5:**
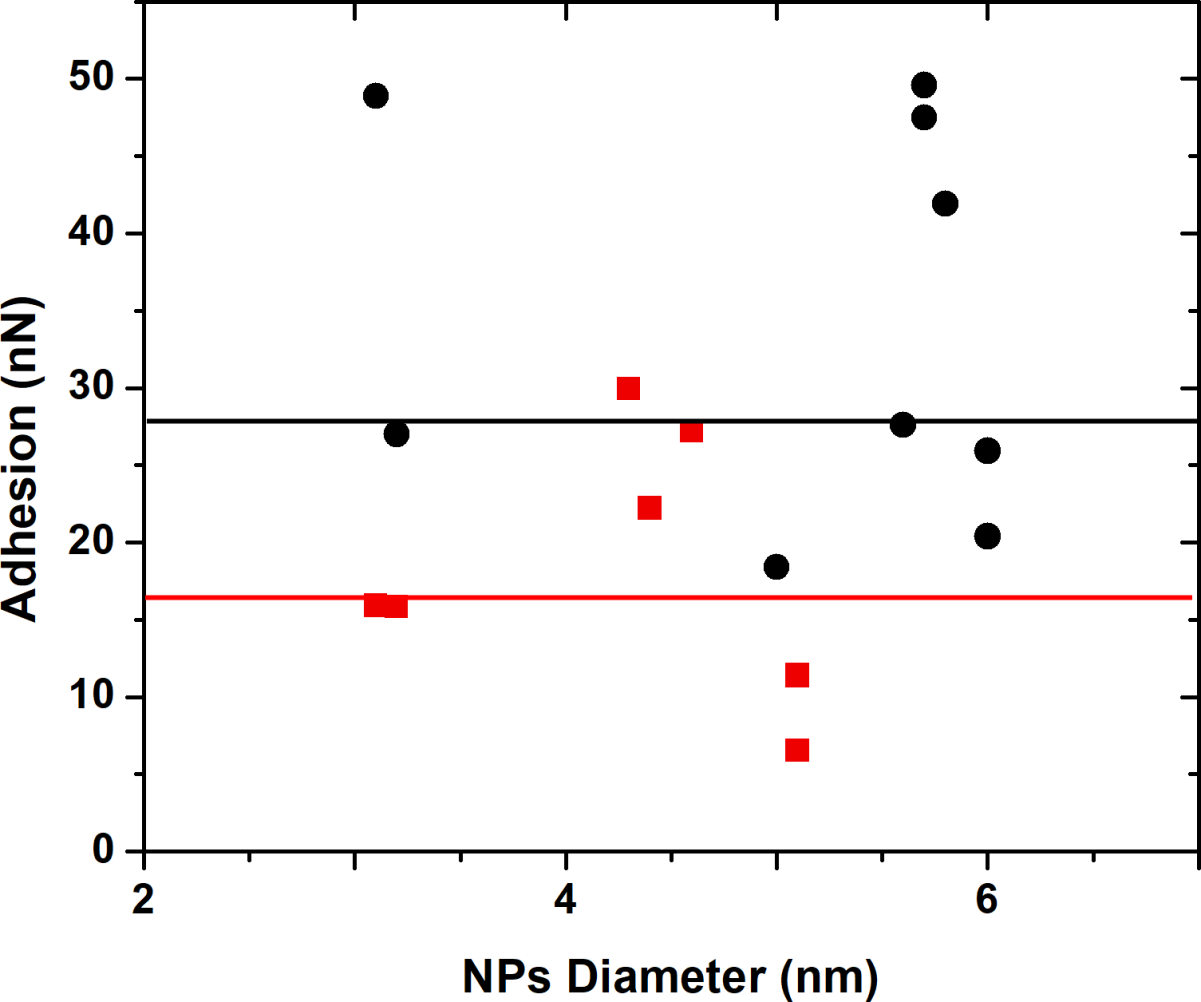
Adhesion forces between silicon AFM probe and nanoparticles covered with –NH_2_ tail group synthesized by different methods resulting in different packing densities: black circle – two-step method, red square – one-step method. Black and red lines represent respectively the mediane values corresponding to the adhesion force between Au NPs synthesized respectively by two-step and one step methods.

The packing density of a thin coating depends also on the functionalization method (one step or two step processes). We have performed additional adhesion measurements on Au NPs functionalized with an amino (–NH_2_) tail group synthesized either in a one-step or a two-step process. The two-step functionalization method has favored a higher adhesion values when compared to values obtained for the one-step method (Figure 5). This result is in accordance with our expectation as a two step process favors a higher SAMs packing density at the surface of the Au NPs contrary to the one step process. In one step synthesis of NPs [42], the NH_2_ functionalized thiol molecule has two roles: 1) reduction of Au ions to produce Au NPs and 2) stabilization of these Au NPs by embedding them with the SAMs of thiols in solution. In the two-step process, Au ions are first reduced by a surfactant such as sodium citrate to obtain 10 nm diameter particle, for instance, and then, the thin layer of citrate molecules is exchanged by the thiol layer: this process is favorable as Au has a stronger affinity with thiol than carboxylic groups of sodium citrate molecules [43]. A two-step process represents a longer method, however by choosing the appropriate functionalization tail group of the molecule, it is possible to properly tune the adhesion of NPs and obtains a highly dense SAMs coating.

### Adhesion on flat Au films

Particle morphology and diameter can affect the value of the adhesion forces between particles in contact with a surface [17]. In order to estimate the effect of particle geometry on the adhesion between functionalized Au and a silicon tip, measurements were repeated on flat 10 nm thick Au films functionalized with –CH_3_, –NH_2_ and –SH tail group molecules. The results of the measurements are displayed in Table 1. For all groups, adhesion measured on flat substrates is approximately 2–3 times higher in comparison to nanoparticles with the same functionalization. This is probably due to a higher ligand density on the surface that allows a better assembly of molecule to form a highly compact thin film [44]. It is important that although the absolute adhesion values measured on the films are different compared to the particles, the trend from least to most adhesive remains similar.

**Table 1:** Average adhesion forces of Au films with methylene (–CH_3_), amino (–NH_2_) and thiol (–SH) functional groups with standard deviation in brackets. The corresponding values obtained for NPs of around 20 nm diameter coated with the same molecules. The films display a similar adhesion dependence on the ligand end groups as the functionalized particles. Thiol end group covered NP is subjected to the highest interaction with silicon probe tip, and methylene group covered NP to the lowest.

Chemical end group		CH_3_	NH_2_	SH

Adhesion force (nN)	on flat substrate	15.2 ± 1.3	31.1 ± 3.7	46.2 ± 4.9
	on nanoparticles	5 ± 0.5	15 ± 2	25 ± 3

We have estimated the effect of the particle size on adhesion by contact mechanics considerations. The dependence of adhesion on the radii of two spheres *R*_1_ and *R*_2_ in contact is given by the following relation [45]:

[5]
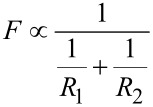


**Figure 6:**
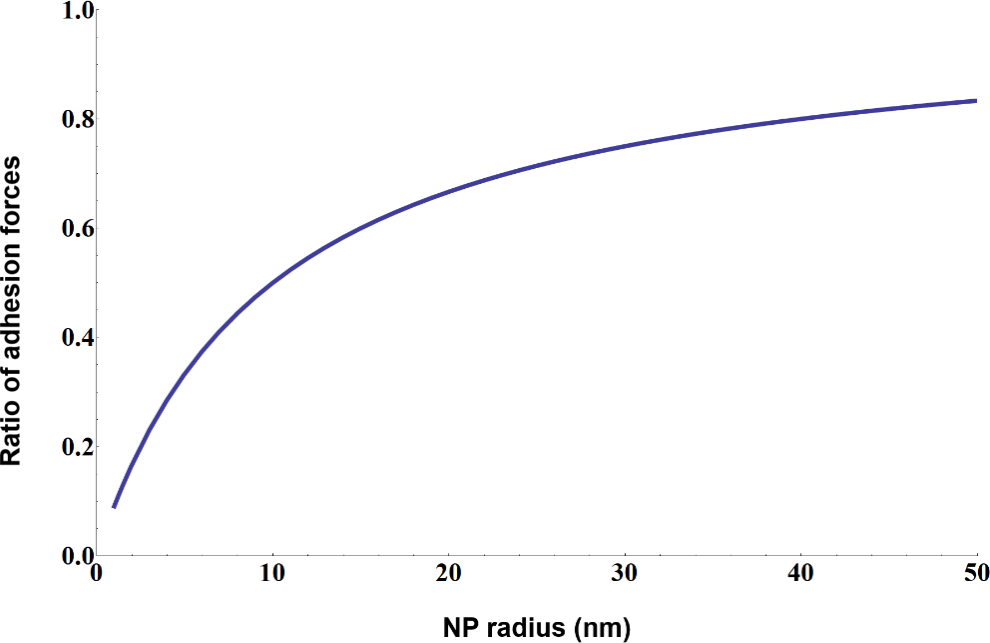
Dependence of adhesion force on the size of two spherical particles in contact. *F*_r_ is the ratio of adhesion force *F*_1_ where *R*_1_ = *R*_2_ = 10 nm and adhesion force *F*_2_ where *R*_1_ = 10 nm and *R*_2_ = 1–50 nm.

Lets assume *R*_1_ to be an AFM tip radius 10 nm and and *R*_2_ to be the particle radius varying in the range from 1 to 50 nm. The corresponding force is *F*_part_. If the AFM tip is in contact with a surface then *R*_2_ is infinite and the corresponding force is *F*_flat_. By plotting the ratio *F*_flat_ to *F*_part_ depending on particle size, the effect of particle diameter on adhesion can be esitmated (Figure 6).

Considering the significant number of factors that influence adhesion, it is difficult to take all aspects into account and give decisive explanation of the obtained results and broad scattering of measured values. Possible reasons may include deviation of NPs geometry from spherical shape, variation of the tip radii between different AFM probes, contamination and wear of the AFM tip during the measurements, contamination of the sample by air-born materials etc. In addition, the exact contact area between tip/nanoparticle is unknown, which complicates quantitative analysis and interpretation. The size effect of nanoscale adhesion is not yet fully understood and is on the way to be seriously investigated and correlated with theoretical studies. Nevertheless, it is clear that functionalization is a powerful method to tune adhesion within a broad range.

## Conclusion

In the present study, we have investigated the influence of functionalization on the adhesion between functionalized Au nanoparticles (NPs) and silicon AFM tip. Adhesion forces were mapped by atomic force microscope equipped with PeakForce QNM mode.

It was shown that the adhesion response was significantly affected by the functional nature of the ligands, packing density of the thin molecular films grafted on the NPs, and by the size of the NPs. Depending on the tail-groups adhesion values varied from 29 to 3 nN in the following order: –SH, –NH_2_, –OCH_3_, –OH, COOH and –CH_3_.

Furthermore, we demonstrated the difference in adhesion on the NPs having the same functional groups, but prepared using different methods (one-step vs two-step process). According to the measurements, two-step process favored higher adhesion. The difference was explained by the fact that two-step process yields higher packing density of molecules on the Au surface.

In addition, it was shown that adhesion on flat substrates is 2–3 times higher than that on particles, indicating the importance of the surface curvature. And yet, the overall trend from the least adhesive to the most adhesive remained the same.

With this work we demonstrated that functionalization of nano-objects represents a powerful method for tuning the adhesion of nanoscale systems.

## Experimental

### Preparation and characterization of Au nanoparticles

#### Bare Au nanoparticles

**NPs diameter below 10 nm.** The procedure was referred to a method described in [42]. One milliliter of 1% HAuCl_4_ was added to 90 mL of water. After stirring for 1 min, 2.0 mL of 38.8 mM sodium citrate was added. Subsequently, 1.0 mL of freshly prepared 0.75% NaBH_4_ in 38.8 mM sodium citrate was added. The resulting colloidal solution was stirred for an additional 5 min and stored in a dark bottle at 4 °C. By varying the amount of NaBH_4_ from 2 down to 0.5 mL, the size of the NPs was varied from 4–5 up to 10 nm diameter. The size of NPs depends on the volume of NaBH_4_ added to the solution.

**Growth of Au NPs from 10 to 30 nm in diameter – seed solution.** The colloidal suspension was made by reduction of an aqueous solution of AuNPs, HAuCl_4_·3H_2_O supplied by Sigma-Aldrich. The suspension was stabilized with sodium citrate (provided by Sigma-Aldrich), which, by reducing HAuCl_4_, imparts the negative charge of the citrate ions to the Au NP surface [46–47].

For example, a solution of particles with a diameter of 15 nm was prepared by adding 3 mL of 1% aqueous HAuCl_4_ to 150 mL of pure H_2_O at 90 °C with vigorous stirring, followed one minute later by the addition of 3 mL of 1% aqueous sodium citrate solution. The solution was stirred for 5 min and then stored at 4 °C until needed [47].

By reducing the amount of sodium citrate from 2 to 0.5 mL, the diameters of the NPs were varied from 10 up to 30 nm. Figure 2a displays transmission electron microscopy (TEM) images of Au NPs of 30 ± 5 nm diameter.

**Growth of Au NPs from 30 to 70 nm in diameter.** Au NPs were synthesized by a seed-mediated process described in [48]. Immediately after the synthesis of the Au seeds (NPs of 15 nm diameter) the same reaction vessel was heated until the temperature of the solution reached 90 °C. Then, 1 mL of a HAuCl_4_ solution (25 mM) was injected. After 30 min, the reaction was finished. This process was repeated twice. After that, 55 mL of the sample was extracted with 53 mL of pure water and 2 mL of 60 mM sodium citrate. This solution was then used as a seed solution, and the process was repeated again until reaching the desired Au NPs diameter. By changing the volume extracted in each growth step, it is possible to tune the seed particle concentration and control the stability of the NPs solution.

#### Coated Au nanoparticles

Au NPs were coated with self-assembled monolayers ending with a hydrophobic (methyl –CH_3_), partially hydrophilic (–OCH_3_) and hydrophilic group (hydroxyl –OH, thiol –SH, carboxylic –COOH, amino –NH_2_). Dodecanethiol for methyl terminated coating (referred to as –CH_3_), 3-(triethoxysilyl)propane-1-thiol for methoxy terminated coating (referred to as –OCH_3_), 2-aminoethanethiol hydrochloride for the amino end group (referred to as –NH_2_), 11-sulfanylundecan-1-ol for the hydroxy tail group (referred to as OH), hexane-1,6-dithiol for the thiol tail group (referred to as –SH) were provided by Sigma-Aldrich and used as received.

Common NPs functionalization methods can be tuned to allow improvements of the modification of NPs surface functionality by choosing the appropriate number of steps: from one step (in situ synthesis) up to several (ex situ), if necessary.

The one-step method described in [49] was applied for the preparation of amine functionalized Au NPs (hydrophilic surfaces). 400 μL of 213 mM 2-aminoethanethiol hydrochloride [C_2_H_7_NS] was added to 40 mL of 1.42 mM HAuCl_4_, and stirred for 30 min at room temperature. 10 μL of 10 mM NaBH_4_ was added under stirring within 30 min in the dark [50]. The solution was stored at 4 °C in a dark bottle. HAuCl_4_ was dispersed in water, reduced by NaBH_4_ and stabilized by C_2_H_7_NS (electrosteric stabilization) due to the thiol–Au bond.

For a hydrophobic and partially hydrophobic coatings, a two-step functionalization method [49] based on a modified version of two common syntheses [51] has been applied. The as-synthesized NPs solution [46–47] was centrifuged at 7,000 rpm for 20 min to pellet the NPs, decanted, and then re-suspended in a proper solvent to reduce the citric acid concentration. The supernatant containing the sodium citrate molecules is then removed and 10 mL of ethanol (for hydrophilic coating) or cyclohexane (for partially and hydrophobic coating) as well as 10 μL of thiol molecules were added under ultrasonication at room temperature for 15 min. The centrifugation first enables to reduce the concentration of citrate molecules (stabilizer agent surrounding the particles) present in the suspension, the solvent (ethanol or cyclohexane) has a dispersant role for the particles and its presence with thiol molecules under sonication enables to exchange citrate molecules physically adsorbed to Au NPs by a covalent binding between thiol and Au NPs surface.

For a hydrophilic coating, the previously used method [47] was slightly modified according to [52] by adding NaOH solution to improve the stability of the colloidal solution. Indeed, a high pH stabilizes the mixture, and prevents agglomeration of the NPs.

The overview of the molecules used for functionalization and obtained chemical end groups is presented in Table 2. NPs were characterized by transmission electron microscopy (TEM, ARM200 from JEOL).

**Table 2:** Overview of all the molecules used to functionalize Au NPs with their respective tail groups – (chemical drawings realized with free MarvinSketch software).

Name	Chemical tail group	Formula

sodium citrate	–COOH	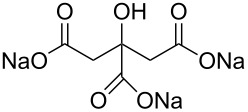
2-aminoethanethiol hydrochloride	–NH_2_	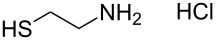
11-sulfanylundecan-1-ol	–OH	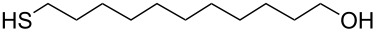
hexadecane-1-thiol	–CH_3_	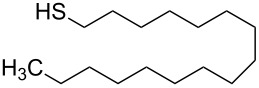
3-(triethoxysilyl)propane-1-thiol	–OCH_3_	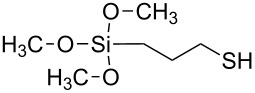
hexane-1,6-dithiol	–SH	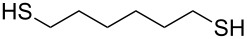

### Drop casting of colloidal solution and drying process

The experimental protocol of drop casting was the following: a microliter drop of gold colloidal solution was deposited onto a cleaned silicon wafer laid flat on a table at 20 °C, allowing nanoparticles to adsorb to the surface during a short time. The drop started to freely dry at room temperature in air (covered by a high beaker to avoid too much air contamination). The samples were characterized by AFM after this drying process.

### Au film preparation

Silicon wafers were cleaned with ultraviolet (UV) light for two hours using a UV lamp with a wavelength of 254 nm (Spectroline, model SCT-1A/F), and then coated with a 10 nm-thick Au film by vacuum coater (model Auto 306, Edwards High Vacuum International). The Au films were functionalized by dipping the Au coated silicon wafers in a solution containing either ethanol for ligands with hydrophilic tail end groups or cyclohexane for ligand molecules with hydrophobic end groups, for 2 h.

### Adhesion measurements

The particles were deposited on silicon wafer by drop-casting. Adhesion was measured by a Bruker Multimode 8 AFM with the Peakforce Quantitative Nanomechanics (PeakForce QNM) mode. PeakForce QNM mode is a recent advancement in AFM method providing quantitative nanomechanical mapping mode with the simultaneous measurement of the sample’s adhesion between tip and sample surface, Young’s modulus (according to either DMT or Sneddon model), deformation and energy dissipation along with the surface topography (Supporting Information File 1).

Etched silicon probes RTESPA-300 with a nominal spring constant *k* ≈ 40 N/m for QNM were provided by Bruker. All used tips were calibrated according to Bruker’s recommendations. During imaging, the setpoint value was set at 10 ± 2 nN, the gain was kept constant between 20 and 25, and the Peakforce amplitude was set at 150. The results were analysed by both Nanoscope analysis software provide by Bruker and Gwyddion.

Gwyddion software was used to extract the average adhesion relative to the particles. The different functions of the data process of the software allows to extract easily the selected area that will be considered for analysis. The bottom of the nanoparticles is not taken into account in the evaluation of the adhesion force average value to avoid any tip artefact measurements. A statistical evaluation on a minimum of 5 different particles has been achieved.

As a first step, a larger area of 3 × 3 µm was mapped to locate the Au NPs. Then, a smaller area containing a few particles of interest was mapped to increase the number of data points obtained on each particle. A total amount of 29 –SH coated Au NPs were analysed, 30 were coated with –NH_2_ terminal group, 34 by –OH, 21 by –OCH_3_, 15 by –COOH and finally 15 by –CH_3_ terminal hydrophobic groups.

## Supporting Information

File 1 PeakForce QNM (Quantitative NanoMechanics).
