# Supplementary material for: Tuning adhesion forces between functionalized gold colloidal nanoparticles and silicon AFM tips: role of ligands and capillary forces
Source: Beilstein J Nanotechnol. 2018 Feb 20;9:660–70. doi: 10.3762/bjnano.9.61 (PMC5827705; doi:10.3762/bjnano.9.61)
Supplement: File 1 — PeakForce QNM (Quantitative NanoMechanics). [file Beilstein_J_Nanotechnol-09-660-s001.pdf]

Supporting Information

for

Tuning adhesion forces between functionalized gold colloidal nanoparticles and silicon AFM tips: role of ligands and capillary forces

Sven Oras*[1,2], Sergei Vlassov[1], Marta Berholts[1,3], Rünno Lõhmus[1] and Karine Mougin[2]

Address: [1]Institute of Physics, University of Tartu, W. Ostwaldi tn 1, 50412, Tartu, Estonia, [2]Université de Strasbourg, Université de Haute Alsace, Institut de Science des Matériaux, IS2M-CNRS-UMR 7361, 15 Rue Jean Starcky, 68057 Mulhouse, France and [3]Department of Physics and Astronomy, University of Turku, FIN-20014 Turku, Finland

Email: Sven Oras - sven.oras@ut.ee

* Corresponding author

PeakForce QNM (Quantitative NanoMechanics)

The PeakForce QNM (Quantitative NanoMechanics) is an extension of Peak Force Tapping Mode ™ developed by Bruker and allows a quantitative measurement of material properties at the nanometer scale; in particular the modulus, the adhesion, the deformation of the material and the energy dissipated during the contact between the tip and the substrate. PeakForce QNM (PFQNM) mode allows mapping complex composite material with a higher resolution than simple Tapping or PeakForce Tapping imagery (≈5 nm) and avoids rapid wear of the tip that taps on the material and potentially could also damage the material. In addition, this mode makes it possible to extract local information relating to the specific properties of the material, thanks to the point-by-point analysis system.

The PFQNM is a new technique based on the real-time analysis of force–distance curves recorded at a frequency of about 2 kHz. As a consequence, the acquisition times for obtaining an image remain reasonable. The principle of PeakForce QNM mode is based on force curve analysis to extract information about tip–substrate adhesion, material modulus, and dissipated energy when tip gets in contact with the substrate. When the approach–withdrawal curves are plotted against time as shown in Figure S1, the signal is close to that of a "Heartbeat" and thus has been called "Heart Beat Curves" by Bruker.

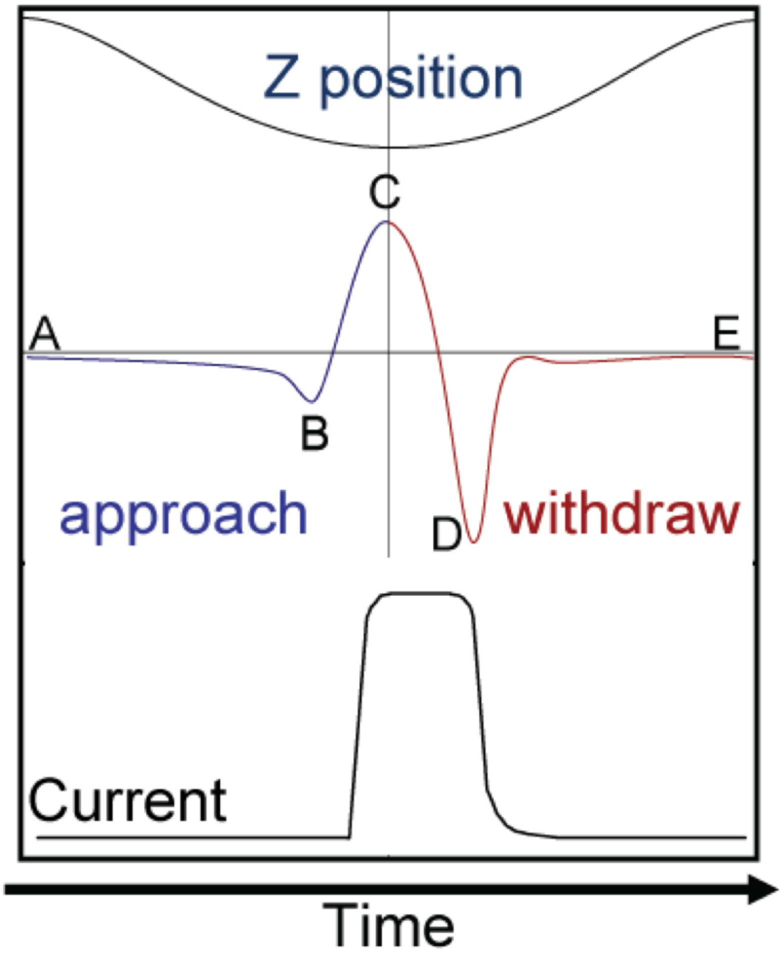

Figure S1: Analysis of forces curves designed as "heartbeat curves" from Bruker' user manual.

The force curve plotted against time (Figure S1) shows the first contact of the probe with the sample (B), the maximum force (C) and the point of adhesion (D). As a function of the Z displacement, these data transformed into a force curve (Figure S2) determine an interaction force between the tip and the surface. Local and real-time mechanical properties of the sample such as adhesion, Young's modulus, energy dissipation, deformation in addition to the surface topography are determined as shown in Figure S2:

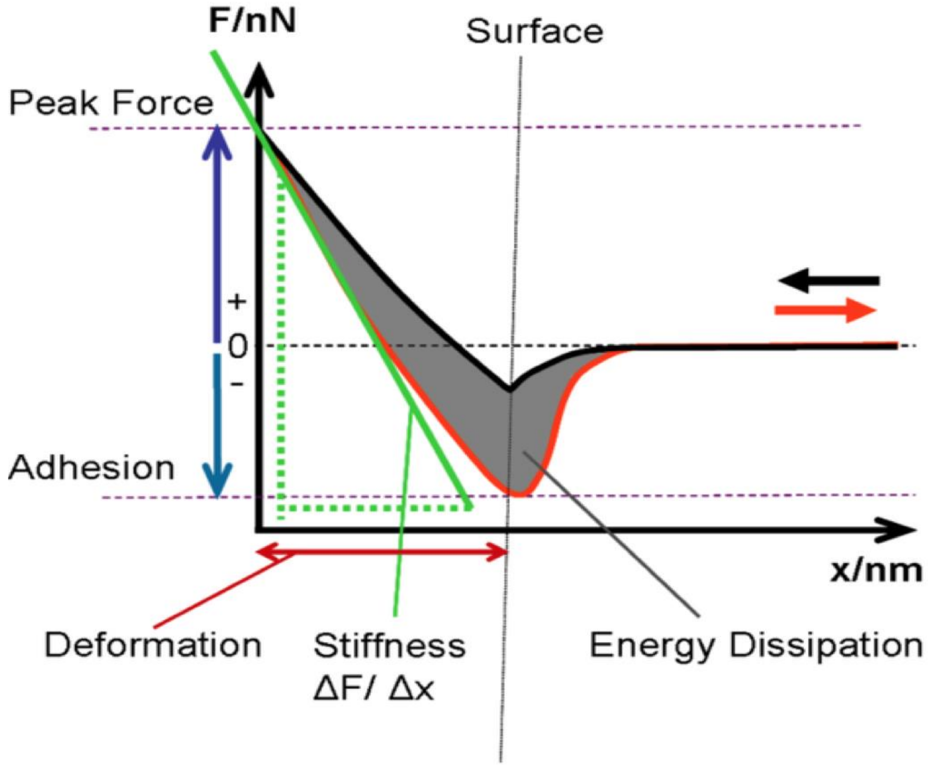

Figure S2: Evaluation of the physico-chemical and mechanical properties from each individual force–distance curve.
